# Impact of supplementation with milk–cereal mix during 6–12 months of age on growth at 12 months: a 3-arm randomized controlled trial in Delhi, India

**DOI:** 10.1093/ajcn/nqab304

**Published:** 2021-10-12

**Authors:** Sunita Taneja, Ravi P Upadhyay, Ranadip Chowdhury, Anura V Kurpad, Himani Bhardwaj, Tivendra Kumar, Pratibha Dwarkanath, Beena Bose, Sarita Devi, Gunjan Kumar, Baljeet Kaur, Rajiv Bahl, Nita Bhandari

**Affiliations:** Centre for Health Research and Development, Society for Applied Studies, New Delhi, India; Centre for Health Research and Development, Society for Applied Studies, New Delhi, India; Centre for Health Research and Development, Society for Applied Studies, New Delhi, India; Department of Physiology, St John's Medical College, Bengaluru, India; Centre for Health Research and Development, Society for Applied Studies, New Delhi, India; Centre for Health Research and Development, Society for Applied Studies, New Delhi, India; Department of Physiology, St John's Medical College, Bengaluru, India; Department of Physiology, St John's Medical College, Bengaluru, India; Department of Physiology, St John's Medical College, Bengaluru, India; Centre for Health Research and Development, Society for Applied Studies, New Delhi, India; Centre for Health Research and Development, Society for Applied Studies, New Delhi, India; Department of Maternal, Newborn, Child and Adolescent Health, World Health Organization, Geneva, Switzerland; Centre for Health Research and Development, Society for Applied Studies, New Delhi, India

**Keywords:** linear growth, infancy, animal source protein, milk–cereal mix, randomized controlled trial, India

## Abstract

**Background:**

A large proportion of infants in low- and middle-income countries are stunted. These infants are often fed complementary foods that are low-quality, primarily in terms of protein and micronutrients.

**Objectives:**

We aimed to test 2 milk–cereal mixes supplemented with modest and high amounts of protein during 6–12 mo of age, compared with no supplementation, for their effect on length-for-age *z* score (LAZ) at 12 mo of age.

**Methods:**

Eligible infants (6 mo plus ≤29 d) were randomly assigned to either of the 2 interventions (modest- and high-protein) or a no supplement group. The milk–cereal mixes provided ∼125 kcal, 30%–45% energy from fats, and 80%–100% RDA of multiple micronutrients (MMN). The modest-protein group received 2.5 g protein [protein energy ratio (PER): 8%; 0.75 g from milk source] and the high-protein group received 5.6 g protein (PER: 18%, 1.68 g from milk source). One packet was given daily for 180 d. Counseling on continued breastfeeding and optimal infant-care practices was provided to all.

**Results:**

We enrolled 1548 infants (high-protein: *n* = 512; modest-protein: *n* = 519; and no supplement: *n* = 517). Compared with the no supplement group, there was an improvement in LAZ [adjusted mean difference (MD): 0.08; 95% CI: 0.01, 0.15], weight-for-age *z* score (MD: 0.12; 95% CI: 0.06, 0.19), weight-for-length *z* score (MD: 0.11; 95% CI: 0.02, 0.19), and midupper arm circumference *z* score (MD: 0.10; 95% CI: 0.02, 0.18) in the high-protein group at 12 mo of age. No significant differences for these anthropometric indicators were noted between the modest-protein and no supplement groups or between the high- and modest-protein groups.

**Conclusions:**

Cereal mixes with higher amounts of milk-based protein and MMN may lead to improvement in linear growth and other anthropometric indexes in infants, compared with no supplementation.

This trial was registered at ctri.nic.in as CTRI/2018/04/012932.

## Introduction

A substantial proportion of under-5 children in India and other low- and middle-income countries (LMICs) are stunted [length-for-age *z* score (LAZ) < −2] (1, 2). Much of this stunting occurs in the first 2 y of life (3, 4). Available data from LMICs show that of the total deficit in length at 2 y of age, approximately one-third is already present at birth, over one-third occurs during the 3- to 11-mo period, and a little less than one-third occurs in the 12- to 23-mo period (4, 5). Childhood stunting has been known to negatively affect cardiometabolic health, intellectual learning, educational attainment, and economic capabilities later in life (6–11).

The benefits of exclusive breastfeeding (EBF) in reducing mortality and morbidity are well documented (12). However, the evidence of its benefits on growth is weak (13). Studies have not found increased rates of EBF to be associated with improved LAZs in the first 24 mo of life (14, 15). With regards to nutritional supplementation in children younger than 24 mo old, the evidence supports a small, yet significant, effect on LAZ (+0.08 SD) and weight-for-length *z* score (WLZ; +0.05 SD), especially in food-insecure populations (16). A recently published network meta-analysis using 79 RCTs involving 81,786 children showed that supplementation with multiple micronutrients (MMN) led to a small improvement in height-for-age *z* score (HAZ) and a modest decrease in stunting among children (17). Further, food supplementation, including small-quantity lipid-based nutrient supplement (SQ-LNS), decreased the risk of stunting but did not show improvements in LAZ (17).

Complementary feeding is usually inadequate in resource-poor populations in LMICs, particularly in the critical period of 6–12 mo of age when declines in linear growth are observed (18–21). The concerns are with both the quantity and quality of complementary foods because infants often fail to achieve the optimal intake of key nutrients required to achieve linear growth. High-quality proteins, micronutrients, and other specific nutrients may be particularly important to achieve optimal linear growth in infancy, because of the additional requirements in LMICs on account of high rates of microbial exposure, infection, and gut inflammation (22, 23). Analysis of complementary foods for 6- to 12-mo-old infants in poor populations in India showed that adequate intake of growth-limiting nutrients such as thiamin, riboflavin, selenium, vitamin B-6, zinc, and phosphorus could not be achieved using home-available foods (24–26).

An important nutrient with a suggested role in promoting growth in children is protein, especially those obtained from animal sources; these have been shown to increase the concentrations of insulin-like growth factor-1 (IGF-1) (27, 28). IGF-1 is an important growth hormone that mediates the linear growth–promoting effect of pituitary growth hormone (GH) (29). It also has a GH-independent growth-stimulating effect and ensures cortical bone integrity. IGF-1 is thought to reduce osteoblast apoptosis and promotes osteoblastogenesis (30–32). This effect leads to increased chondral plate growth. Animal source proteins also have a much higher digestibility and consequent indispensable amino acid (IAA) bioavailability and potentially higher postprandial plasma IAA concentrations than plant source protein (33, 34). **Supplemental Figure 1** provides a conceptual framework through which supplementation with protein may promote linear growth in children.

It is unclear whether increasing the amount of total protein and high-quality protein, particularly from dairy sources, will improve linear growth more substantially. It is desirable that ∼10%–15% of the total daily protein intake for infants and young children should be from animal sources (35). In many settings, however, young children derive proteins largely from plant sources (36). It is yet unknown whether a relatively higher yet safe intake of protein, particularly from animal sources, is better than the currently recommended intake in accelerating linear growth (37). This randomized controlled trial aimed at evaluating 2 nutritional supplements with varying amounts of protein in 6- to 12-mo-old infants, compared with a control group that received counseling but no food supplementation, for their effect on linear growth at 12 mo of age.

## Methods

### Study setting, design, and participants

An individually randomized controlled efficacy trial (CTRI/2018/04/012932) was conducted in low-resource settings in urban Delhi, India. Study participants were infants aged 6 mo (plus ≤29 d).

### Screening and enrolment

A door-to-door survey was conducted in the urban neighborhoods of Delhi by the survey team to identify infants aged 6 mo (plus ≤29 d). Infants aged <6 mo were followed up periodically until they became 6 mo of age. The screening and enrolment team visited the home, explained the study to the mother and other family members, and screened the infant for eligibility. For inclusion in the study, infants had to be aged 6 mo (plus ≤29 d), breastfed, with no documented illness requiring prolonged institutional management, not severely malnourished (weight-for-height < −3 SD), and with no major congenital malformations, and the family had to be unlikely to relocate from the study area over the next 6 mo. If the infant was eligible, consent for participation was obtained from the primary caregiver (usually the mother). Group allocation was requested through a Web-based system. Socioeconomic characteristics of the family were documented. Anthropometric measurements [length, weight, midupper arm circumference (MUAC), and head circumference (HC)] were obtained.

### Randomization, allocation, and blinding

Infants were randomly assigned to 1 of the groups—modest-protein supplement, high-protein supplement, or no supplement—through a randomization list prepared using blocks of variable (3 and 6) length. The allocation ratio followed was 1:1:1. The list was prepared by a statistician, based at the WHO, Geneva, Switzerland, who was not otherwise involved with the study. Only 1 infant was enrolled per household. The milk–cereal mix packets were labeled with 13 letters each to maintain team blinding between the modest- and high-protein groups. The list of letters was provided to the company who manufactured these mixes by the WHO statistician. The blinding of the study participants and the outcome ascertainment teams to the group allocation (i.e., no supplement compared with the 2 supplement groups) could not be ensured. However, labeling of the milk–cereal mix packets using different letters maintained participant blinding between the modest- and high-protein groups.

### Study interventions

The 2 intervention groups (modest-protein and high-protein) received packets of milk–cereal mix—1 packet to be consumed daily for a period of 180 d. The modest-protein supplement provided ∼125 kcal; 2.5 g protein [protein energy ratio (PER): 8%]; 30% of the total protein from milk sources (0.75 g); 30%–45% energy from fats; and 80%–100% RDA of growth-relevant MMN (vitamins A, D, C, E, B-12, B-6, B-1, and B-2, niacin, pantothenic acid, biotin, zinc, calcium, selenium, iodine, magnesium, manganese, and copper) (38). The high-protein supplement provided ∼125 kcal; 5.6 g protein (PER: 18%); 30% (1.68 g) of the total protein from milk sources; 30%–45% energy from fats; and 80%–100% RDA of micronutrients. **Supplemental Table 1** provides the nutritional details of the supplements.

We aimed to provide ∼50%–60% of the non-breast-milk energy requirement through the supplement (39). The milk–cereal mixes were in the form of 25-g sachets, prepared by Pristine Organics Pvt. Ltd. (https://pristineorganics.com/) located in Bangalore, India. These were available with the following ingredients: rice and pulses; wheat and apple; rice and banana; and rice and mixed fruits. The cereal mixes were pretested for acceptability in infants in the study population before study initiation.

In the groups receiving supplement, cereal mixes of the mother's choice were provided, with an option for her to change her preference at the time of weekly replenishment. To prevent sharing, cookies (called biscuits in the Indian context) were provided for other children in the household. Infants in the control group did not receive any supplement. The supplement delivery team visited households weekly to provide milk–cereal sachets. They gathered information on compliance by collecting empty packets and reinforced their intake. Subjects with low compliance were visited by the team supervisor to resolve queries of the families and discuss barriers to optimal intake.

Mothers of infants in all 3 study groups were counseled by nutritionists on the importance of continuing breastfeeding and on appropriate complementary feeding practices using home foods. Mothers were also taught early recognition of illness and counseled on early care-seeking and on the importance of childhood vaccines. Iron–folic acid (IFA) drops (Ferrium XT, Emcure Pharmaceuticals) were provided to all infants enrolled in the study as per WHO recommendations (40). Mothers were advised to give 1 mL of the syrup daily, which provided 10 mg of elemental iron and 100 μg folic acid. Bottles were replenished fortnightly.

### Sample size

We assumed a 0.20-SD (0.55 cm, 1 SD = 2.74 cm) (5) mean difference (MD) of LAZ at 12 mo between the modest-protein group and the no supplement group and a 0.30-SD (0.82 cm, 1 SD = 2.74 cm) difference between the high-protein group and the no supplement group. With 80% power, 2-sided 5% α level, and 10% attrition, 430 infants and 190 infants each were required for the comparisons of the modest-protein and high-protein groups with the no supplement group, respectively. We, therefore, aimed to enroll a total of 1290 infants. Further, with a sample size of 430 infants each in the modest-protein and high-protein groups, we expected to detect a 0.20-SD difference in LAZ between the 2 supplement groups.

Based on the observation of a higher than assumed (∼15%–20%) loss to follow-up due to outmigration, the investigators approached the Technical Advisory Group (TAG) constituted for the study. The TAG recommended increasing the sample size by 20% to ensure adequate statistical power. The sample size was, therefore, revised to 516 in each of the 3 groups, i.e., a total of 1548 infants.

### Outcomes and their ascertainment

The primary outcome was attained LAZ at 12 mo of age. The secondary outcomes were change in LAZ and WLZ between 6–9 and 9–12 mo of age; the proportion stunted (LAZ < −2) and wasted (WLZ < −2) at 12 mo of age; and mean MUAC *z* score (MUAC-Z) and mean HC *z* score at 12 mo of age. Additional secondary outcomes were the proportion breastfed, mean hemoglobin concentration, and the proportion with anemia at 12 mo of age.

Outcome ascertainment was by an independent team, who were kept unaware of the group allocation to the maximum extent possible. Weights and lengths were measured by a pair of workers using digital weighing scales (model 354; Seca) and infantometers (model 417; Seca) to the nearest 10 g and 0.1 cm, respectively. HC and MUAC measurements were taken using measuring tapes (model 212; Seca). Inter- and intraobserver standardization exercises for anthropometric measurements were conducted at study initiation and at 3-mo intervals thereafter. Information on breastfeeding was taken for all, and 24-h dietary recalls were performed in a subsample at infant age 12 mo by nutritionists. Continued breastfeeding was defined as the mother still breastfeeding. Data on morbidities (fever, pneumonia, diarrhea, and hospitalization) were collected for the preceding 2 wk from the time of visit at 9 and 12 mo of age. Blood samples at 12 mo were collected at home by trained phlebotomists. Anemia assessment was done using capillary blood via a HemoCue Hb 201+ analyzer (41). Infants with hemoglobin concentrations <11 g/dL were considered to have anemia (42). All study staff received training in good clinical practice guidelines.

### Statistical analysis

Analyses were conducted using STATA version 16.0 (StataCorp LLC, College Station, TX, USA). Means ± SDs or medians [IQRs] were calculated for continuous variables and proportions for categorical variables. A comparison of means, medians, and proportions by the 3 study groups was done to check whether the randomization scheme resulted in the groups being comparable. For each infant, compliance to the milk–cereal mix was presented as a percentage. This was calculated as the total number of sachets consumed divided by the number of sachets that should have been consumed (i.e., 180 sachets), multiplied by 100. We calculated means and SEs of intakes of energy, carbohydrates, protein, and fats from 24-h dietary recalls for each of the 3 groups.

The primary analysis was the comparison of outcomes between the 3 study groups. The comparisons were between the following groups: *1*) the modest-protein compared with the no supplement group; *2*) the high-protein compared with the no supplement group; and *3*) the modest-protein compared with the high-protein group. For continuous outcomes, a generalized linear model (GLM) of the Gaussian family with an identity-link function was used to calculate the effect size (difference in means and 95% CIs). For binary outcomes, a GLM of the binomial family with a log-link function was used to calculate the effect size (RRs and 95% CIs). Purposive selection of variables for adjustment in the model was made; those that brought ≥15% change in the univariate effect size between study groups and outcome were considered for adjustment. Based on this, for the primary outcome, i.e., attained LAZ at 12 mo of age, adjustment in the model was performed for LAZ and weight-for-age *z* score (WAZ) at the time of enrolment and mother's education.

We also developed a GLM of the Gaussian family with an identity-link function for changes in anthropometric indexes from enrollment to 9 mo and from 9 to 12 mo. We followed the same principles as we used in the primary analyses for the selection of variables for multivariable analysis. We also built generalized estimating equation (GEE) models to estimate the effect of infant supplementation with milk–cereal mix over the 6 mo of the intervention delivery period. This approach accounted for interdependence between multiple measurements in the same infant and ensured that infants with data at any of the time points were included in the analysis (intention-to-treat principle). We used GEE models of the Gaussian family with an identity-link function, an autoregressive covariance-variance matrix taking time into account, and robust SEs. The models were adjusted for baseline LAZ, WAZ at enrollment, and mother's years of education.

For all analyses, effect sizes were reported with 95% CIs. A *P* value < 0.05 was considered for statistical significance. We created graphs for the means of LAZ, WAZ, and WLZ at 9 and 12 mo of age according to intervention groups using the lowess smoothing technique.

### Ethics approval and consent to participate

The study was approved by the ethics committee of the Centre for Health Research and Development, Society for Applied Studies, India (SAS/ERC/IMPRINT-I/2018). Written informed consent was obtained in the local language from the caregivers before enrolment.

## Results

**FIGURE 1:**
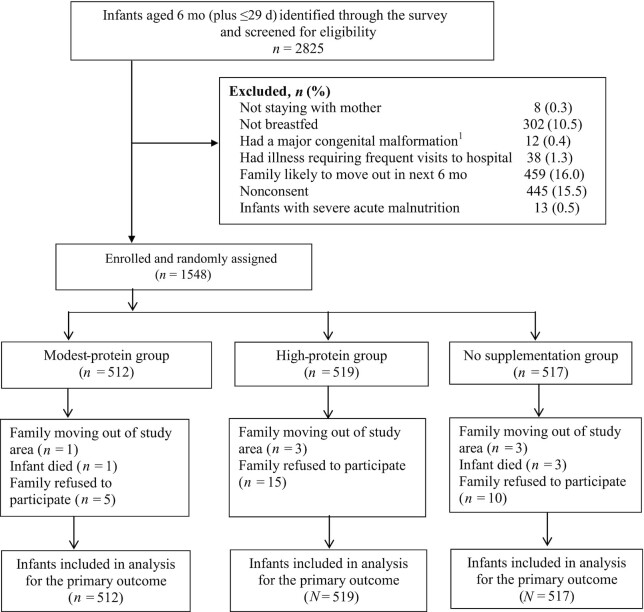
Trial profile. ^1^Major congenital malformations included cardiac (3), skeletal/limb (7), and oral cavity (2).

Between 2 July, 2018 and 10 July, 2019, a total of 2825 infants aged 6 mo (plus ≤29 d) were identified through the survey and screened for eligibility. We excluded 1277 infants because they did not meet eligibility criteria, or the family did not give consent to participate (**Figure 1**). A total of 1548 infants were enrolled and randomly assigned to 1 of the 3 groups, i.e., the modest-protein (*n* = 512), the high-protein (*n* = 519), and the no supplement (*n* = 517) group (Figure 1).

There were no statistically significant differences between the groups in terms of baseline characteristics (**Table 1**). In the modest-protein, high-protein, and no supplement groups, the mean infant age at enrolment was 6.5 ± 0.2 mo, 6.6 ± 0.2 mo, and 6.5 ± 0.2 mo, respectively; mean weight was 6.93 ± 0.9 kg, 6.93 ± 0.9 kg, and 6.89 ± 0.9 kg, respectively; mean LAZ was −1.19 ± 1.0, −1.16 ± 1.1, and −1.18 ± 1.0, respectively; and mean WAZ was −1.10 ± 1.0, −1.08 ± 1.1, and −1.16 ± 1.0, respectively (Table 1). The proportion of infants stunted at enrolment was 21.9% in the modest-protein group, 20.1% in the high-protein group, and 19.8% in the no supplement group. The maternal age in the modest-protein, high-protein, and no supplement groups (mean ± SD: 24.8 ± 3.9 y, 25.2 ± 3.9 y, and 25.1 ± 4.0 y, respectively) and years of schooling (8 [4–10], 8 [3–10], and 8 [3–10], respectively) were similar in all 3 groups (Table 1).

**TABLE 1:** Baseline characteristics of the enrolled infants and their families, by study group^1^

	Intervention		
	Modest-protein group (*n* = 512)	High-protein group (*n* = 519)	No supplement group (*n* = 517)
Infant characteristics at enrolment			
Age at enrolment, mo	6.5 ± 0.2	6.6 ± 0.2	6.5 ± 0.2
Males	258 (50.4)	245 (47.2)	267 (51.6)
Weight,^2^ kg	6.93 ± 0.9	6.93 ± 0.9	6.89 ± 0.9
Length,^2^ cm	64.91 ± 2.4	64.90 ± 2.6	64.92 ± 2.5
LAZ using WHO standards	−1.19 ± 1.0	−1.16 ± 1.1	−1.18 ± 1.0
Stunted (< −2 LAZ)	112 (21.9)	104 (20.1)	102 (19.8)
WLZ using WHO standards	−0.41 ± 1.0	−0.41 ± 1.1	−0.50 ± 1.0
Wasted (< −2 WLZ)	28 (5.5)	40 (7.7)	28 (5.4)
WAZ using WHO standards	−1.10 ± 1.0	−1.08 ± 1.1	−1.16 ± 1.0
Underweight (< −2 WAZ)	97 (19.0)	93 (18.0)	108 (20.9)
MUAC, cm	13.5 ± 1.0	13.5 ± 1.1	13.4 ± 1.0
MUAC-Z	−0.58 ± 0.9	−0.56 ± 1.0	−0.64 ± 0.9
Head circumference, cm	41.1 ± 1.3	41.1 ± 1.4	41.1 ± 1.3
HC *z* score	−1.61 ± 1.0	−1.59 ± 1.0	−1.58 ± 0.9
Socio-demographic characteristics			
Wealth quintile			
Poorest	103 (20.1)	101 (19.5)	106 (20.5)
Very poor	100 (19.5)	96 (18.5)	114 (22.1)
Poor	113 (22.1)	95 (18.3)	101 (19.5)
Less poor	92 (18.0)	114 (22.0)	104 (20.1)
Least poor	104 (20.3)	113 (21.7)	92 (17.8)
Annual family income, USD	2467 [1644–3289]	2467 [1644–3399]	2467 [1644–3426]
Nuclear family	243 (47.5)	249 (47.9)	253 (48.9)
Religion: Hindu	417 (81.5)	414 (79.8)	402 (77.8)
Maternal characteristics			
Age, y	24.8 ± 3.9	25.2 ± 3.9	25.1 ± 4.0
Duration of schooling, y	8 [4–10]	8 [3–10]	8 [3–10]
Never been to school	107 (20.9)	121 (23.3)	110 (21.3)
Home makers	485 (94.7)	488 (94.0)	487 (94.2)
Paternal characteristics			
Age, y	28.4 ± 4.6	28.9 ± 4.5	28.7 ± 4.6
Duration of schooling, y	8 [5–10]	8 [5–10]	8 [5–11]
Unemployed	7 (1.4)	14 (2.7)	9 (1.7)

1 Values are mean ± SD, median [IQR], or *n* (%). HC, head circumference; LAZ, length-for-age *z* score; MUAC, midupper arm circumference; MUAC-Z, midupper arm circumference *z* score; WAZ, weight-for-age *z* score; WLZ, weight-for-length *z* score.

2 Data not available for 2 infants.

**Table 2** presents the data on compliance to the milk–cereal mix and IFA among the 3 groups. The mean days a packet of milk–cereal mix was consumed, over the 6-mo intervention period, by infants in the modest- and high-protein groups was 158.2 ± 29.4 and 154.4 ± 34.6, respectively. The proportion of infants who consumed milk–cereal mix on >75% of days was 85.6% for the modest-protein group and 82.9% for the high-protein group. The mean days IFA was consumed by infants was 151.2 ± 33.1 for the modest-protein group, 148.1 ± 37.2 for the high-protein group, and 146.5 ± 32.5 for the no supplement group. The proportion of infants who consumed IFA for >75% of days was 74.4%, 71.1%, and 66.3% for the modest-protein, high-protein, and no supplement group, respectively.

**TABLE 2:** Compliance to the infant milk–cereal mix and IFA^1^

	Intervention		
Indicators of compliance	Modest-protein group (*n* = 512)	High-protein group (*n* = 519)	No supplement group (*n* = 517)
Days supplement packet consumed by the infant	158.2 ± 29.4	154.4 ± 34.6	—
Days packet consumed, %			
>75	438 (85.6)	430 (82.9)	—
51–75	54 (10.6)	64 (12.3)	—
26–50	14 (2.7)	17 (3.3)	—
≤25	6 (1.2)	8 (1.5)	—
Days IFA syrup consumed by the infant	151.2 ± 33.1	148.1 ± 37.2	146.5 ± 32.5
Days IFA consumed, %			
>75	381 (74.4)	369 (71.1)	343 (66.3)
51–75	99 (19.3)	110 (21.2)	139 (26.9)
26–50	26 (5.1)	30 (5.8)	28 (5.4)
≤25	6 (1.2)	10 (1.9)	7 (1.4)

1 Values are mean ± SD or *n* (%). IFA, iron–folic acid.

The mean LAZ at 12 mo in the modest-protein, high-protein, and no supplement groups was −1.45 ± 1.0, −1.38 ± 1.0, and −1.49 ± 1.1, respectively. Compared with the no supplement group, there was an improvement in LAZ among infants from the high-protein group (adjusted MD: 0.08; 95% CI: 0.01, 0.15) (**Table 3**). No significant differences in LAZs were noted in comparisons between the modest-protein and no supplement groups or between the modest- and high-protein groups.

**TABLE 3:** Effect of infant supplementation with milk–cereal mix on growth, breastfeeding, and biochemical outcomes at 12 mo of age^1^

				Adjusted risk ratio or adjusted MD (95% CI)^2^		
	Modest- protein group (*n* = 512)	High-protein group (*n* = 519)	No supplement group (*n* = 517)	Modest-protein group vs. no supplement group (ref.)	High-protein group vs. no supplement group (ref.)	High-protein group vs. modest-protein group (ref.)
Attained anthropometric measures at 12 mo of age^3^						
Primary outcome						
LAZ (*n* = 498, 496, 496)	−1.45 ± 1.04	−1.38 ± 1.02	−1.49 ± 1.09	0.04 (−0.03, 0.11)	0.08 (0.01, 0.15)^4^	0.04 (−0.03, 0.11)
Secondary outcomes						
WAZ (*n* = 498, 496, 496)	−1.28 ± 1.00	−1.20 ± 0.97	−1.39 ± 1.02	0.06 (−0.01, 0.13)	0.12 (0.06, 0.19)^4^	0.06 (−0.01, 0.13)
WLZ (*n* = 498, 496, 496)	−0.78 ± 0.98	−0.72 ± 0.94	−0.89 ± 0.97	0.05 (−0.03, 0.14)	0.11 (0.02, 0.19)^4^	0.05 (−0.04, 0.14)
MUAC-Z (*n* = 498, 496, 496)	−0.71 ± 0.92	−0.65 ± 0.92	−0.80 ± 0.93	0.04 (−0.04, 0.12)	0.10 (0.02, 0.18)^4^	0.06 (−0.02, 0.14)
HC *z* score (*n* = 498, 496, 496)	−1.50 ± 0.96	−1.56 ± 0.99	−1.60 ± 0.90	0.07 (−0.03, 0.17)	0.02 (−0.09, 0.12)	−0.06 (−0.16, 0.05)
Stunted (*n* = 498, 496, 496)	139 (27.9)	135 (27.2)	147 (29.6)	0.96 (0.76, 1.21)	0.90 (0.71, 1.14)	0.94 (0.74, 1.20)
Wasted (*n* = 498, 496, 496)	58 (11.7)	48 (9.7)	63 (12.7)	1.04 (0.72, 1.48)	0.75 (0.51, 1.09)	0.72 (0.49, 1.06)
Underweight (*n* = 498, 496, 496)	118 (23.7)	93 (18.8)	145 (29.2)	0.90 (0.70, 1.15)	0.64 (0.49, 0.83)^4^	0.69 (0.53, 0.91)^4^
MUAC <12.5 cm (*n* = 498, 496, 496)	54 (10.8)	43 (8.7)	63 (12.7)	0.98 (0.68, 1.41)	0.67 (0.45, 0.98)^4^	0.69 (0.46, 1.03)
MUAC-Z < −2 (*n* = 498, 496, 496)	39 (7.8)	28 (5.7)	51 (10.3)	0.89 (0.59, 1.37)	0.52 (0.33, 0.83)^4^	0.58 (0.35, 0.95)^4^
HC *z* score < −2 (*n* = 498, 496, 496)	151 (30.3)	152 (30.7)	162 (32.7)	0.97 (0.78, 1.21)	0.96 (0.77, 1.20)	0.99 (0.79, 1.24)
Change in anthropometric measures^2,3^						
Change in LAZ (6–9 mo) (*n* = 488, 484, 483)	−0.05 ± 0.52	−0.01 ± 0.56	−0.09 ± 0.53	0.03 (−0.04, 0.09)	0.08 (0.01, 0.14)^3,4^	0.05 (−0.01, 0.11)
Change in LAZ (9–12 mo) (*n* = 488, 485, 484)	−0.19 ± 0.53	−0.20 ± 0.50	−0.20 ± 0.50	0.00 (−0.06, 0.07)	0.00 (−0.07, 0.06)	0.00 (−0.07, 0.06)
Change in WLZ (6–9 mo) (*n* = 488, 484, 483)	−0.25 ± 0.69	−0.25 ± 0.74	−0.27 ± 0.65	0.04 (−0.04, 0.12)	0.04 (−0.04, 0.11)	−0.01 (−0.09, 0.07)
Change in WLZ (9–12 mo) (*n* = 488, 485, 484)	−0.10 ± 0.65	−0.04 ± 0.68	−0.11 ± 0.68	0.02 (−0.06, 0.10)	0.07 (−0.01, 0.15)	0.05 (−0.04, 0.13)
Change in MUAC, cm (6–9 mo) (*n* = 483, 482, 480)	0.16 ± 0.65	0.21 ± 0.62	0.09 ± 0.64	0.07 (−0.01, 0.16)	0.12 (0.04, 0.20)^4^	0.05 (−0.03, 0.13)
Change in MUAC, cm (9–12 mo) (*n* = 488, 485, 484)	0.05 ± 0.61	0.05 ± 0.63	0.09 ± 0.63	−0.04 (−0.11, 0.04)	−0.03 (−0.11, 0.04)	0.00 (−0.07, 0.08)
Hb concentration and proportion with anemia						
Hb, g/dL (*n* = 485, 484, 490)	10.4 (1.19)	10.4 (1.18)	10.3 (1.29)	0.15 (−0.01, 0.30)	0.15 (−0.01, 0.30)	0.00 (−0.16, 0.15)
Proportion anemic (*n* = 485, 484, 490)	318 (65.6)	304 (62.8)	342 (69.8)	0.94 (0.81, 1.09)	0.90 (0.77, 1.05)	0.96 (0.82, 1.12)
Breastfeeding						
Proportion with continued breastfeeding (*n* = 504, 501, 500)	469 (93.1)	468 (93.4)	472 (94.4)	0.98 (0.86, 1.12)	0.99 (0.87, 1.12)	1.01 (0.89, 1.14)

1 Values are *n* (%) and means ± SDs, with outcome measures of adjusted risk ratio for proportion with stunting, wasting, underweight, MUAC < 12.5 cm, MUAC-Z < −2, HC *z* score < −2, anemia, and continued breastfeeding; and with adjusted MD for other parameters. We used a GLM of the Gaussian family with an identity-link function for continuous outcomes; GLM of the binomial family with a log-link function for binary outcomes; and a GLM of the Gaussian family with an identity-link function for changes in anthropometric indexes from enrollment to 9 mo and from 9 to 12 mo. GLM, generalized linear model; Hb, hemoglobin; HC, head circumference; LAZ, length-for-age *z* score; MD, mean difference; MUAC, midupper arm circumference; MUAC-Z, midupper arm circumference *z* score; WAZ, weight-for-age *z* score; WLZ, weight-for-length *z* score.

2 Adjusted for LAZ, WAZ score at enrolment, and mother's years of education for anthropometric outcomes; no adjustments were made for Hb concentration (g/dL), proportion with anemia, and proportion with continued breastfeeding.

3 Infants with any of the following at 9 and/or 12 mo of age (denoting extreme values or implausible data points based on familiarity with the study population) were excluded from the growth-based analysis: LAZ < −6 or LAZ >2; WAZ < −6 or WAZ >2; or WLZ < −5 or WLZ >2. A total of 17 infants were therefore excluded.

4 Statistically significant at *P* < 0.05.

Similarly, for WAZ, WLZ, and MUAC-Z, compared with the infants from the no supplement group, those in the high-protein group had improved WAZ (adjusted MD: 0.12; 95% CI: 0.06, 0.19), WLZ (adjusted MD: 0.11; 95% CI: 0.02, 0.19), and MUAC-Z (adjusted MD: 0.10; 95% CI: 0.02, 0.18) at 12 mo of age. There were no significant differences in the comparisons between the modest-protein and no supplement groups as well as for comparisons between the high- and modest-protein groups with regards to WAZ, WLZ, and MUAC-Z at 12 mo of age (Table 3).

**FIGURE 2:**
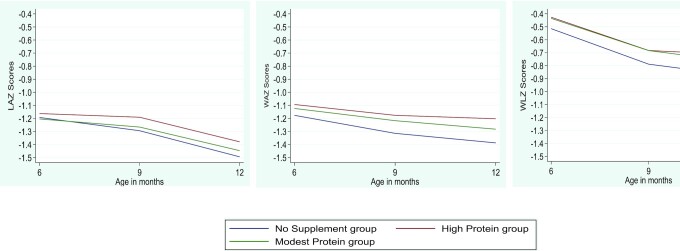
Infant anthropometric measures (LAZ, WLZ, WAZ) from 6 to 12 mo of age, by study group. The figure was created using the lowess smoothing technique. Numbers used to construct the graphs: no supplement group, *n* = 483; high-protein group, *n* = 484; modest-protein group, *n* = 488. LAZ, length-for-age *z* score; WAZ, weight-for-age *z* score; WLZ, weight-for-length *z* score.

Compared with the no supplement group and modest-protein group, the risk of being underweight (WAZ < −2) was 36% (RR: 0.64; 95% CI: 0.49, 0.83) and 31% (RR: 0.69; 95% CI: 0.53, 0.91) lower in infants in the high-protein group, respectively. The risk of having a MUAC-Z < −2 was 48% lower in infants in the high-protein group as opposed to the no supplement group (RR: 0.52; 95% CI: 0.33, 0.83) and 42% lower than in the modest-protein group (RR: 0.58; 95% CI: 0.35, 0.95) (Table 3). Compared with the no supplement group, infants in the high-protein group had a significant change in LAZ (adjusted MD: 0.08; 95% CI: 0.01, 0.14) and MUAC (cm) (adjusted MD: 0.12; 95% CI: 0.04, 0.20) between 6 and 9 mo (Table 3). There were no significant differences among infants in the 3 study groups for mean hemoglobin concentration, proportion anemic, and proportion with continued breastfeeding. **Figure 2** presents the trajectory of change in anthropometric measures (LAZ, WLZ, and WAZ) from 6 to 12 mo of infant age across the 3 study groups.

Similar findings were obtained from the GEE-based analysis. Compared with the no supplement group, there was an improvement in LAZ among infants from the high-protein group (adjusted MD: 0.07; 95% CI: 0.01, 0.13) over the 6-mo intervention period (**Table 4**). No significant differences in LAZ were noted in comparisons between the modest-protein and no supplement groups or between the modest- and high-protein groups. Similarly, for WAZ, WLZ, and MUAC, compared with the infants from the no supplement group, those in the high-protein group had improved WAZ (adjusted MD: 0.09; 95% CI: 0.04, 0.15), WLZ (adjusted MD: 0.07; 95% CI: 0.003, 0.14), and MUAC (adjusted MD: 0.09; 95% CI: 0.01, 0.17) over the 6 mo of the intervention period (Table 4).

**TABLE 4:** Effect of infant supplementation with milk–cereal mix on anthropometric measures during the 6-mo intervention period using a GEE model^1^

	Modest-protein group vs. no supplement group (ref.)	High-protein group vs. no supplement group (ref.)	High-protein group vs. modest-protein group (ref.)
LAZ	0.03 (−0.03, 0.09)	0.07 (0.01, 0.13)^2^	0.05 (−0.01, 0.11)
WAZ	0.06 (0.00, 0.11)	0.09 (0.04, 0.15)^2^	0.04 (−0.02, 0.10)
WLZ	0.05 (−0.02, 0.12)	0.07 (0.003, 0.14)^2^	0.02 (−0.05, 0.10)
MUAC	0.05 (−0.03, 0.13)	0.09 (0.01, 0.17)^2^	0.04 (−0.04, 0.13)

1 Values are adjusted MDs (95% CIs). MDs were calculated by using GEEs of the Gaussian family with an identity-link function, an autoregressive covariance-variance matrix taking time into account, and robust SEs and adjusted for time, LAZ, WAZ score at enrolment, and mother's years of education. GEE, generalized estimating equation; LAZ, length-for-age *z* score; MD, mean difference; MUAC, midupper arm circumference; WAZ, weight-for-age *z* score; WLZ, weight-for-length *z* score.

2 Statistically significant at *P* < 0.05.

The findings from the 24-h dietary recalls among 150 infants suggested significant differences in the energy and protein consumed among infants from the high-protein group when compared with the no supplement group (energy: MD: 135.1 kcal; 95% CI: 20.7, 249.5 kcal; protein: MD: 6.0 g; 95% CI: 2.0, 10.1 g). There were also significant differences in the protein consumed between infants from the high-protein and modest-protein groups (MD: 4.3 g; 95% CI: 0.6, 8.0 g) (**Table 5**). There were no significant differences in the proportion of infants with morbidities across the 3 groups at 9 and 12 mo of age (**Table 6**).

**TABLE 5:** Twenty-four-hour dietary recalls in enrolled infants at 12 mo of age^1^

	Modest-protein group (*n* = 50)	High-protein group (*n* = 50)	No supplement group (*n* = 50)
Energy,^2^ kcal	468.9 ± 38.9	524.1 ± 42.5	389.0 ± 38.9
Protein,^3^ g	13.3 ± 1.1	17.6 ± 1.5	11.5 ± 1.4
Protein energy ratio, %	11.3	13.4	11.8
Fat, g	12.6 ± 1.6	15.1 ± 2.0	13.9 ± 1.9
Carbohydrate, g	54.2 ± 4.9	63.1 ± 5.6	52.9 ± 4.9

1 Values are means ± SEs unless indicated otherwise. ANOVA was used to compare means ± SEs between the 3 groups.

2 Statistically significant difference between the high-protein and no supplement groups.

3 Statistically significant difference between the high-protein and no supplement groups; and between the high- and modest-protein groups.

**TABLE 6:** Data on reported morbidity and hospitalizations among infants enrolled in the study^1^

	Intervention		
	Modest-protein group (*n* = 512)	High-protein group (*n* = 519)	No supplement group (*n* = 517)
Morbidity at 9 mo (*n* = 488, 485, 494)			
Pneumonia^2^	6 (1.2)	3 (0.6)	5 (1.0)
Severe pneumonia^3^	—	—	—
Diarrhea^4^	66 (13.5)	65 (13.4)	58 (11.7)
Fever^5^	131 (26.8)	128 (26.4)	127 (25.7)
Hospitalized since last visit	1 (0.2)	2 (0.4)	1 (0.2)
Morbidity at 12 mo (*n* = 505, 501, 501)			
Pneumonia^2^	7 (1.4)	4 (0.8)	7 (1.4)
Severe pneumonia^3^	2 (0.4)	—	—
Diarrhea^4^	54 (10.7)	67 (13.4)	65 (13.0)
Fever^5^	140 (27.7)	126 (25.2)	143 (28.5)
Hospitalized since last visit	4 (0.8)	2 (0.4)	3 (0.6)

1 Values are *n* (%). Data on morbidity collected for the last 2 wk from the time of data collection; chi-square test was used to compare proportions. No statistically significant differences in proportions (at *P* < 0.05) between the 3 groups were noted for the morbidity outcomes considered.

2 History of cough (as reported by the mother or caregiver) or difficulty breathing and reported fast breathing or chest indrawing.

3 Pneumonia with general danger signs: not able to breastfeed, feed, or drink; lethargy; unconsciousness; or stridor.

4 Diarrhea (as reported by the mother or caregiver) with or without symptoms of dehydration. Symptoms of dehydration: not able to drink, lethargy; unconsciousness, restlessness, or irritability; and sunken eyes.

5 Fever as reported by the mother or caregiver.

## Discussion

Our study found that daily supplementation for 180 d, starting from 6 mo of age, with milk–cereal mix having a higher amount of protein (5.6 g) with added MMN resulted in improvements in infant LAZ, WAZ, WLZ, and MUAC-Z at 12 mo of age, compared with the group that received no supplement. No significant improvements in growth outcomes were noted in those receiving milk–cereal mix with a modest amount of protein (2.5 g) and MMN, compared with the no supplement group. The risk of being underweight and having MUAC-Z < −2 was lower in infants receiving cereal mix with higher protein than in both the no supplement group infants and those receiving cereal mix with a modest amount of protein (2.5 g). We noted no significant differences among infants in the 3 study groups for mean hemoglobin concentration, proportion anemic, and proportion being breastfed.

These findings are similar to a recent trial involving infants aged 6–12 mo who received either SQ-LNS [each 20-g packet: 114 kcal energy; 3.0 g protein (PER: 10%; ∼0.6 g milk source protein)], SQ-LNS-plus [each 20-g packet: 113 kcal energy; 3.7 g protein (PER: 13%; ∼1.6 g milk source protein)], or no supplementation (43). Both SQ-LNSs contained micronutrients and essential fatty acids. A small positive effect of supplementation with SQ-LNS-plus on LAZ at 8 mo (MD: 0.11; 95% CI: 0.01, 0.22) and 10 mo (MD: 0.16; 95% CI: 0.04, 0.27) was seen, but not at 12 mo of age (MD: 0.09; 95% CI: −0.02, 0.21), compared with no supplementation (43). The high-protein with added MMN supplement provided in our study was similar to the SQ-LNS-plus and we observed similar effect sizes for LAZ. Although we noted a small positive effect on LAZ with use of this cereal mix compared with no supplementation, we were unable to detect a significant effect on the proportion of stunted children, probably because the study was not adequately powered.

We did not find a significant improvement in growth parameters between the modest-protein and the no supplement groups. One possible reason could be that the modest-protein group did not provide the protein amount necessary for promotion of growth. Even the dietary assessments in a subsample of infants showed that the total energy, fat, and carbohydrate intakes were similar in the 2 intervention groups. Intake of high amounts of protein, particularly those from animal sources, is especially important in children from low-resource settings because they often have high infection load and poor gut health characterized by gut inflammation and immune activation (22, 23, 44, 45). In such situations, requirements of high-quality proteins, micronutrients, and other specific nutrients may increase. Another important issue to note is that the supplement provided 125 kcal of energy, i.e., for the 6- to 9-mo-old, this equated to 60%, and for the 9- to 12-mo-old, it equated to 40% of the non-breast-milk energy requirements. During the period of 6–12 mo, the LAZ decreased in all groups with a steep decline after 9 mo of age, which may have been due to the insufficient energy provided through the supplements. Studies investigating the effect of nutritional supplementation during the complementary feeding period on growth of children have met with varied results (46, 47). Possible reasons driving this diversity in findings could be differences in the nutrient composition of these supplements, the duration of supplementation, and the follow-up period, with some assessing linear growth at 18–24 mo of age (46, 47).

Currently, there is insufficient evidence on how the effect of supplementation on child growth is affected by quality of protein. Some studies, mostly observational, have shown that consumption of animal-based protein, especially meat, is associated with improved linear growth and reduced risk of overweight (48–51). Mechanisms linking protein intake with child growth are also not understood adequately. Few studies have shown that supplementation with animal source protein is linked to increased concentrations of insulin and IGF-1 (27, 28). IGF-1 is one of the growth factors for bone growth; however, a link between increased concentrations and child growth has not been conclusively established (29–31). Available literature suggests that the gut microbiome could influence child growth (52, 53). The research in this area is still exploratory and the effects of supplementation with different types of protein-rich foods on the gut environment are still unclear. Krebs et al. (54) in their study among 6- to 9-mo-old infants found that those supplemented with high-protein, meat-based diets had a higher proportion of SCFAs producing gut bacteria than those on low-protein, cereal-based diets. In another study, protein intake during the early complementary feeding period was associated with increased gut microbiome diversity as well as abundance of SCFAs producing gut bacteria (55). Emerging evidence points to the role of SCFAs in regulating activation of G-protein couple receptors, which in turn, regulates fat accumulation and energy expenditure (56). Further, these fatty acids have been shown to regulate bone metabolism, particularly reducing bone loss due to inflammation (57).

The strengths of this study include a rigorous design and outcome assessments by a trained and standardized team. Our study had a few limitations. First, owing to the nature of the intervention, it was not possible to ensure complete blinding, with respect to the 3 groups, for the study outcome ascertainment team as well as for the participants. However, blinding was ensured between the 2 intervention groups. Although we attempted to reduce the bias due to lack of blinding between the intervention and control groups by having different study teams for intervention delivery and outcome assessments, we acknowledge that this strategy might not have removed bias introduced before the outcomes were measured. Second, whereas direct observation of consumption of the milk–cereal mixes would have been ideal, an assessment of compliance was reported. Third, this study had limited power to detect a small effect size. We did not have adequate power to detect a significant difference in mean LAZ between the high- and modest-protein groups at 12 mo of age. Lack of any significant difference in the primary outcome between these 2 groups could therefore be due to low power.

We did not assess biomarkers of protein status and, therefore, were unable to conclusively tease out whether the lack of significant impact of supplementation was due to inadequate protein intake or protein utilization.

In conclusion, supplementation during the second half of infancy, for a period of 180 d, with a cereal mix having a higher quantity of milk-based protein with added MMN leads to improvement in linear growth and other anthropometric indexes (weight-for-age, weight-for-length, and MUAC) in children from low-resource settings, compared with no supplementation. Complementary feeding programs may consider providing foods with high-quality, particularly milk-based, protein and MMN. However, the increase in the cost of the supplements due to increased protein content will need to be considered, especially when planning for a large-scale rollout.

## Supplementary Material

nqab304_Supplemental_File

## Data Availability

The organization conducting the trial (Society for Applied Studies, India) is a collaborator in the Healthy Birth, Growth, and Development Knowledge Integration (HBGDKi) of the Bill & Melinda Gates Foundation and the data generated from the study will be shared as part of the HBGDKi repository (https://github.com/HBGDki). However, individual requests will be considered on a case-to-case basis. The request for data should be accompanied by a detailed proposal describing the scientific questions to be addressed. Proposals should be submitted to ST (sunita.taneja@sas.org.in).

## Notes

Supported by Bill & Melinda Gates Foundation grant OPP1177843 through the Biotechnology Industry Research Assistance Council of the Department of Biotechnology, Government of India (to ST). The funders had no role in the study design, data collection or analysis, decision to publish, or preparation of the manuscript.

Supplemental Figure 1 and Supplemental Table 1 are available from the “Supplementary data” link in the online posting of the article and from the same link in the online table of contents at https://academic.oup.com/ajcn/.

Abbreviations used: EBF, exclusive breastfeeding; GEE, generalized estimating equation; GH, growth hormone; GLM, generalized linear model; HC, head circumference; IAA, indispensable amino acid; IFA, iron–folic acid; IGF-1, insulin-like growth factor-1; LAZ, length-for-age *z* score; LMIC, low- and middle-income country; MD, mean difference; MMN, multiple micronutrients; MUAC, midupper arm circumference; MUAC-Z, midupper arm circumference *z* score; PER, protein energy ratio; SQ-LNS, small-quantity lipid-based nutrient supplement; TAG, Technical Advisory Group; WAZ, weight-for-age *z* score; WLZ, weight-for-length *z* score.
