# Supplementary material for: Impact of supplementation with milk–cereal mix during 6–12 months of age on growth at 12 months: a 3-arm randomized controlled trial in Delhi, India
Source: Am J Clin Nutr. 2021 Oct 12;115(1):83–93. doi: 10.1093/ajcn/nqab304 (PMC8754995; doi:10.1093/ajcn/nqab304)
Supplement: nqab304_Supplemental_File [file nqab304_supplemental_file.doc]

“Impact of supplementation with milk-cereal mix during 6 to 12 months of age on growth at 12 months: a three-arm randomized controlled trial in Delhi, India”. Sunita Taneja et al.

**ONLINE SUPPLEMENTARY MATERIAL**

**Supplementary Table 1. Nutrient composition of the two milk cereal mixes used in the study**

|  | **Composition of milk cereal mix (Modest protein group)** | **Composition of milk cereal mix**  **(High protein group)** |
| --- | --- | --- |
| **Nutrients** | **Per Sachet (In 25 gms)** | **Per Sachet (In 25 gms)** |
| Energy (kcal) | 126.5 | 126.9 |
| Protein (g) | 2.5 | 5.6 |
| Fat (g) | 5.5 | 5.5 |
| Carbohydrate (g) | 16.75 | 13.75 |
| Vitamin A (µg) | 400 | 400 |
| Vitamin D (µg) | 5 | 5 |
| Vitamin E (mg) | 2.7 | 2.7 |
| Ascorbic Acid (mg) | 30 | 30 |
| Thiamine (mg) | 0.3 | 0.3 |
| Riboflavin (mg) | 0.4 | 0.4 |
| Niacin (mg) | 6.6 | 6.6 |
| Pantothenic acid (mg) | 1.8 | 1.8 |
| Pyridoxine (mg) | 0.3 | 0.3 |
| Vitamin B12 (µg) | 0.7 | 0.7 |
| Zinc (mg) | 6.6 | 6.6 |
| Biotin (µg) | 6 | 6 |
| Calcium (mg) | 100 | 100 |
| Iodine (µg) | 90 | 90 |
| Potassium (mg) | 160 | 160 |
| Phosphorus (mg) | 90 | 90 |
| Magnesium (mg) | 54 | 54 |
| Selenium (µg) | 10 | 10 |
| Copper (µg) | 220 | 220 |
| Manganese (mg) | 0.06 | 0.06 |

**Supplementary Figure 1. Proposed pathways through which animal protein supplementation may influence infant growth**

Increase Insulin

Increase Insulin like growth factor-1

Activation of rapamycin complex C1 (mTORC1)

High Quality Protein or Animal Protein

Chondral plate growth

Casein

Whey

Improve linear growth
